# White or Woke Christian Nationalists? How Race Moderates the Link Between Christian Nationalism and Progressive Identities

**DOI:** 10.1093/poq/nfaf009

**Published:** 2025-05-13

**Authors:** Samuel L Perry, Allyson F Shortle, Eric L McDaniel, Joshua B Grubbs

**Affiliations:** Sam K. Viersen Presidential Professor, Department of Sociology, University of Oklahoma, Norman, OK, US; Associate Professor, Department of Political Science, University of Oklahoma, Norman, OK, US; Professor, Department of Political Science, University of Texas at Austin, Austin, TX, US; Associate Professor, Department of Psychology, University of New Mexico, Albuquerque, NM, US

## Abstract

Scholarship on “Christian nationalism” often frames it as antithetical to progressive politics. Yet recent studies find that historically disadvantaged racial minorities often espouse more progressive political views as Christian nationalism increases. Building on an understanding that American religion and politics are fundamentally racialized and drawing on nationally representative data from a nonprobability sample with a Christian nationalism scale incorporating ideology and self-identification, we examine how racial identity moderates the link between Christian nationalism and how much Americans identify with the terms “woke” and “progressive.” Results reveal racial divergence. As Christian nationalism increases, White Americans are either no different or less likely to affirm progressive identities, while Black Americans become more likely to identify as “woke,” and both Black and Hispanic Americans become more likely to identify as “progressive.” Patterns are also consistent across partisan identity. Results further affirm how race moderates Christian nationalist views and demonstrate how endorsing progressive identities is differentially shaped by race and religion.

Discourse around “Christian nationalism,” understood as an ideology that idealizes and advocates a fusion of Christianity with American civic life, often frames the construct as antithetical to progressive politics (Whitehead and Perry 2020). This is likely because most scholarly attention to the topic has documented how Christian nationalist views (operationalized in various ways) are associated with conservative ideological identities and Republican partisanship (Braunstein and Taylor 2017; Djupe, Lewis, and Sokhey 2023; Vegter, Lewis, and Bolin 2023) as well as ethnocentric, authoritarian, and even antidemocratic attitudes and behaviors (McDaniel, Nooruddin, and Shortle 2011, 2022; Armaly, Buckley, and Enders 2022; Gorski and Perry 2022). Indeed, Djupe, Lewis, and Sokhey (2023, p. 27) go so far as to call Christian nationalism essentially “a Republican project,” given how it so consistently aligns with Republican views.

Yet, Christian nationalism’s documented, contentious relationship to progressivism lends itself to the false impression that Christian nationalist beliefs hold a *universally* adversarial relationship to progressive politics. To be sure, White Americans who subscribe to Christian nationalism are almost invariably found to embrace more reactionary views (Armaly, Buckley, and Enders 2022; Gorski and Perry 2022; Perry et al. 2022). Yet several studies show that as Black and Hispanic Americans more strongly affirm the same measures on surveys, they tend to hold more progressive views on issues of religious diversity, racial inequality, and ethnoracial inclusion (Perry and Whitehead 2019; Perry et al. 2024a, 2024b). They may also be more likely to support Democratic candidates (McDaniel, Nooruddin, and Shortle 2022).

In light of these findings, the current study examines how racial identity potentially moderates the link between Christian nationalism and whether Americans embrace more progressive social identities. Unlike explanations for racial injustice or views about diversity or inclusion, identities tap into something more fundamental to human social relationships, and indeed, are often the source from which Americans’ political, religious, and racial views emerge (Achen and Bartels 2016; Mason 2018). Research on group identities in intergroup interactions suggests our social identities or self-conceptions drive our shared opinions about issues affecting in-group and out-group members (Bardon 2020; Perry 2023, 2024). Thus, examining how the association between Christian nationalism and Americans’ politically salient social identities potentially varies across racial identities helps us better understand not only the racialized nature of American religion, and Christian nationalism specifically, but how political identities themselves are differentially related to Americans’ religious and racial characteristics.

Drawing on original survey data from a nationally representative nonprobability sample of Americans (N = 1,771) and utilizing a measure of Christian nationalism that incorporates previously tested ideological measures with strong face validity as well as a measure of self-identification, we predict the extent to which Americans identify with the terms “woke” and “progressive” and how this association varies by race. We find support for our hypothesis that racial identity moderates the link between Christian nationalism and self-identification with progressive identities. Predictably, among White Americans, Christian nationalism is either unassociated or negatively associated with affirming progressive identities. However, as Christian nationalism increases among Black Americans, the more likely they are to identify with “woke,” and among Black and Hispanic Americans, both become more likely to identify with “progressive.” Our findings thus extend our understanding of Christian nationalism as a construct as well as the racially situated and contingent nature of politically salient social identities.

Before proceeding with our analyses, the following sections frame our expectations in light of research on Christian nationalism and racial identity as well as how racial and religious background differentially shape political views and identities.

## Background

### Christian Nationalism and Racial Identity

Though definitions of “Christian nationalism” can vary slightly (likely due to the explosion of research on the topic across social science and humanities fields), there is general consensus about the term across sociologists and political scientists. Scholars typically have in mind a set of beliefs and narratives that idealize a “deep story” about America’s mythic relationship with “Christianity” (e.g., Gorski and Perry 2022; see also Braunstein 2021; McDaniel, Nooruddin, and Shortle 2011, 2022) and the need to (re)institutionalize “Christian” supremacy in the present day (Whitehead and Perry 2020; Armaly, Buckley, and Enders 2022; Gorski and Perry 2022; Djupe, Lewis, and Sokhey 2023; Vegter, Lewis, and Bolin 2023). Yet the dominant scholarly understanding of Christian nationalism is largely shaped in reference to its most visible—largely White evangelical or charismatic—proponents, who have in mind privileging a particular ethno-religious expression of “Christianity.” This focus may cause scholars to ignore the possibility of different ethno-religious understandings of a “Christian nation” and its implications for divergent political views.

Unlike the “civil religion” Bellah (1967) described among America’s political leaders, which was nonsectarian and united Americans under ideas of providence, justice, and stewardship, Christian Right leaders in decades past (e.g., Falwell 1980; Kennedy 2003) and contemporary activists promoting “Christian nationalism” by name (e.g., Torba and Isker 2022; Wolfe 2022) have a specific ethno-religious tradition in mind. The “Christianity” they envision is of the Anglo Protestant/White Evangelical tradition. It is not that of social justice–oriented Black Protestants, Latino Roman Catholics, or gender-egalitarian and LGBT-affirming Mainline Protestants (Braunstein 2021). This underscores the fact that “religion” nearly always stands for more than orthodox doctrines, but rather is a set of overlapping social identities and assumptions about who “we” are as a group (Wilde and Glassman 2016; Perry 2023; Al-Kire et al. 2024). Given its most visible representation, scholars largely frame “Christian nationalism” neither as a form of “civil religion,” nor as an ecumenical movement to elevate “Christianity” per se, but an ideology that privileges the power of a group with overlapping (and today, threatened) social identities, namely, native-born, White, conservative, Protestant men (Al-Kire et al. 2021; Gorski and Perry 2022; Walker and Haider-Markel 2024a, 2024b).

Yet survey research shows that racial minorities often affirm indicators of Christian nationalism or its similar constructs at rates roughly identical to those of White Americans (Delehanty, Edgell, and Stewart 2019; Whitehead and Perry 2020; Gorski and Perry 2022; McDaniel, Nooruddin, and Shortle 2022; Djupe, Lewis, and Sokhey 2023). Understanding how American religion, and Christian nationalism in particular, is fundamentally shaped by racial identity and racial group interest requires scholars to explore how affirming Christian nationalist measures might mean different things for different Americans. Some studies of Christian nationalism find that race does not moderate Christian nationalism’s link to all political attitudes (e.g., Whitehead and Perry 2020; Djupe, Lewis, and Sokhey 2023), which is most often the case in situations where the outcome in question does not involve issues directly related to ethnic or racial group position or interest, such as LGBT or gender issues (Whitehead and Perry 2020). Most others, however, find a moderating influence of racial identity on how Christian nationalism corresponds to views on racial inequality or religious and ethnoracial exclusion (for surveys of this research, see Perry 2022, 2023).

Several of these studies have focused on the Black-White binary. Perry and Whitehead (2019), for example, find that White Americans who equate being Christian with being “truly American” are more likely to blame Black Americans for racial inequality, while their Black counterparts become more likely to blame discrimination. Other studies find that the more White Americans subscribe to Christian nationalist beliefs, the more they are more likely to feel that White people are victims of discrimination and deny anti-Black police injustice, while Black Americans do not become more likely to hold such views (Perry et al. 2022; Gorski and Perry 2022).

Moving beyond the Black-White binary, Perry and Schleifer (2023) find that while White Americans who conflate Christian and American identities are more likely to believe Americans should support their country even when it is in the wrong, their Black and Hispanic counterparts do not subscribe to such “blind patriotism” as Christian nationalism increases. And recently, Perry et al. (2024a, 2024b) find that Black Americans who affirm Christian nationalism become more likely to view religious diversity as a national strength, and that when their own ethnic or racial group is in view, Black and Hispanic Americans who hold Christian nationalist views become more supportive of cultural pluralism and inclusion. This is all in contrast with White Americans, who consistently oppose religious and racial diversity and inclusion as their adherence to Christian nationalism increases. McDaniel, Nooruddin, and Shortle (2022) even find that Black and Hispanic Americans who in past years affirmed a divinely appointed role for the United States became more supportive of Obama. This all suggests that a key dynamic within Christian nationalist ideology is sacralizing and promoting the status of “our people,” or our ethno-religious in-group, helping us understand why it often correspondents to divergent attitudes across ethnic and racial groups. Below, we consider this as it potentially relates to progressive identities.

### Theorizing Christian Nationalism and Progressive Social Identities

Taken together, research suggests that Christian nationalism is generally articulated by scholars as a set of beliefs that justify the status quo, which supports the maintenance of White (conservative) Christians’ power in the national hierarchy. Yet emerging research also demands a more inclusive examination of Christian nationalism’s diverse theological interpretations. We argue that Christian nationalism can explain a wider range of political attachments for a diversity of Americans, depending on factors such as social group positionality and whether certain values or groups are implicated in a political discussion (McDaniel, Nooruddin, and Shortle 2022; Perry et al. 2024a). In this section, we develop specific expectations related to Christian nationalism, racial identity, and identification with more progressive social identities.

Though evidence has clearly shown that the White image of a Christian nation is linked to identification with conservative partisan and ideological identities (Gorski and Perry 2022), this may not be the case for Black or Hispanic Americans. Research has repeatedly demonstrated, for example, that while White and Black Americans may use the same political language, it does not lead to similar policy and partisan preferences (Dawson 2001; Harris-Lacewell 2004; McDaniel 2009; Philpot 2017; Jefferson 2020). This is also apparent when looking at the connections between religious beliefs and political attitudes (McDaniel and Ellison 2008; McKenzie and Rouse 2013). The highly segregated nature of American religious and political life historically meant that while groups may have used the same labels, they took on different meanings (McDaniel 2009; Jefferson 2020).

For White Americans, political and religious discourse has been characterized by their position as dominant group members, with discussions focusing on how to either protect or promote their dominance (Williams 2020). Conversely, for Black Americans, their religious and political experiences reflect their social position as an oppressed group trying to find a way to survive and hopefully thrive in a harsh and dangerous environment (Raboteau 2001). Because of this, the development of Black political and religious life was focused on freedom and justice for Black Americans (McDaniel 2009; McRoberts 2020). In Lincoln and Mamiya’s (1990) seminal work on the Black church, they put forward the concept of the Black Sacred Cosmos, which represents the distinctiveness of the Black religious experience in America. Viewed through the Black Sacred Cosmos, religious salvation and freedom from physical bondage were often inextricably linked. Because of this, the image of a “Christian nation” in the Black religious experience is a nation in which oppressed races and classes, and particularly Black Americans, experience full equality.^1^ Further, unlike their White counterparts who view the nation as the pinnacle of God’s creation, Black Americans are more likely to view it as a work in progress (Thurman 1976; Phillips 2018; McRoberts 2020). All this suggests that Christian nationalist views, for Black Americans, would intuitively incline them to embrace identities that are associated with liberation, progress, and freedom for Black Americans rather than rejecting such identities as equated with moral decline or cultural “liberalism.”

The connection between Christian nationalism, Hispanic Americans, and political views and identities has received far less attention, certainly compared to White Americans, but also Black Americans as well. This leads our expectations to be slightly more tentative. As noted earlier, McDaniel, Nooruddin, and Shortle (2022) found that Hispanic Americans who subscribed to “American religious exceptionalism” were more likely to support Obama. And those who most ardently believe America has been granted divine purpose are more likely to identify as Democrats compared to those who hold such beliefs more loosely. And importantly, Perry et al. (2024b) find that the more Hispanic Americans connect being “truly American” with being Christian, the more likely they are to affirm inclusive and tolerant stances toward immigrants. This suggests that, like Black and White Americans, when the target group in question is perceived to be “like us,” Christian nationalism likely connects identities to whatever positions benefit those groups. Building on this idea, we expect that Christian nationalist views would incline Hispanic Americans to affirm identities that that are associated with elevation of their in-groups.

## Methods

### Data

Data for this study come from Waves 1 and 3 of the National Addiction and Social Attitudes Survey (NASAS).^2^ The NASAS was a completely web-based survey designed by the authors and fielded by YouGov, an international research data and analytics company. Wave 1 was fielded March 17–April 6, 2022, and Wave 3 was fielded September 26–October 17, 2022. Wave 1 is the source of our control variables, while Wave 3 supplies our Christian nationalism measure and three outcome variables.^3^ Surveys varied in length, but all took under 25 minutes to complete, and key items for this study were asked at the end of Wave 3. YouGov recruits a panel of respondents through websites and banner ads.^4^ These respondents are not paid directly but are entered into lotteries for monetary prizes. Survey invitations were sent to eligible participants and the survey advertised as a general survey on personality, beliefs, and behavior. All consenting documents discussed questions about personality, beliefs, and behavior. Participants were not repeatedly recontacted. In order to draw a nationally representative sample, YouGov employs a method called “matching.” For the general population sample, the frame was constructed by stratified sampling from the full 2019 American Community Survey (ACS) one-year sample with selection within strata by weighted sampling with replacements (using the person weights on the public use file). The cases were weighted to the sampling frame using propensity scores. The matched cases and the frame were combined, and a logistic regression was estimated for inclusion in the frame. The propensity score function included age, gender, race/ethnicity, years of education, and region. The propensity scores were grouped into deciles of the estimated propensity score in the frame and poststratified according to these deciles. The weights were then poststratified on 2016 and 2020 presidential vote choice, and a four-way stratification of gender, age (four categories), race (four categories), and education (four categories), to produce the final weight. It is worth noting that initial demographic information from panel participants is updated every three to six months. Because of the specific recruitment and sampling design, YouGov provides a cooperation rate, which was 87.6 percent at the baseline. The resulting original March 2022 survey sample included 4,363 Americans that were matched and weighted. Attrition between Waves 1 and Wave 3 was primarily due to contracting for fewer follow-up respondents at the third wave, resulting in a total Wave 3 sample of 2,808 respondents, of which our key questions were randomly asked of a subsample of 1,775 respondents. Our final analytic sample in full models is 1,770–1,771 cases after a modest amount of item-level missing data, which were handled with listwise deletion. (Comparisons between the original and analytic sample on key demographic factors showed very little change, suggesting attrition was largely at random; see Supplementary Material table S1.)

### Outcome Variables

Respondents in Wave 3 were given a series of identities and asked, “How well do the following words describe you?” (screenshots of how these identities appeared to respondents are presented in Supplementary Material figure S1). The outcomes for this study are measures assessing how well Americans felt the terms “woke” and “progressive” described them. Responses included 1 = Not at all, 2 = Not too well, 3 = Somewhat well, 4 = Very well, and 5 = Undecided. We recoded values so that “Undecided” is the middle category.^5^ Because the outcomes each have five values, we estimate models with ordinary least squares (OLS) regression.

### Key Independent Variables

The key independent variables for this study are Christian nationalism and racial identity. Christian nationalism has been measured in a variety of ways (McDaniel, Nooruddin, and Shortle 2011; Braunstein and Taylor 2017; Gorski and Perry 2022; Djupe, Lewis, and Sokhey 2023; Perry et al. 2024a, 2024b). We introduce a measure of Christian nationalism incorporating both ideology and self-identification.^6^ Seeking to advance previous measurement efforts from the Baylor Religion Surveys (see Whitehead and Perry 2020), we sought to use ideological measures with strong face validity as capturing the extent to which Americans are fusing Christianity and national heritage and identity. Each measure had been utilized in previous data collection efforts (e.g., Perry and Whitehead 2019; Gorski and Perry 2022; McDaniel, Nooruddin, and Shortle 2022). In addition to other questions about religion and politics (see screenshots of how these questions appeared in Supplementary Material figure S1), we asked respondents, “Please indicate your level of agreement with the following statements”: (1) “America holds a special place in God’s plan.” (2) “The federal government should declare the United States a Christian nation.” (3) “I consider founding documents like the Declaration of Independence and the US Constitution to be divinely inspired.” and (4) “I consider being a Christian an important aspect of being truly American.” As Supplementary Material figure S1 shows, respondents were also asked to indicate how well the term “Christian nationalist” described them. As with the outcome variables, we recoded responses to range from 1 = Not at all to 5 = Very well.^7^ The alpha for these measures is 0.909, indicating high reliability.^8^ We combined items into an additive index from 0 to 20.^9^ Higher scores indicate greater agreement with Christian nationalism.

Racial identity was measured with a series of dichotomous dummy variables from Wave 1, including White (reference), Black, Hispanic, and Other Race. For the multivariate analyses, we also include interaction terms for Christian nationalism and Black, Hispanic, and Other Race Americans. We also test separate models by racial identity and party identity.

### Control Variables

Each analysis includes controls for demographic, political, and religious characteristics from Wave 1. Age is measured in years from 20 to 97. Sex was measured with dummy-coded variables with women (reference), men, and nonbinary persons. Educational attainment was measured with attainment categories from 1 = less than high school to 6 = postgraduate work. Household income is measured with a series of dummy variables, including less than $30K per year, $30–$60K, $60–$100K, $100–$200K, $200K or more, and did not indicate income. We also included a control for living in the southern United States.

Political characteristics include party identity and ideological identity. Party identity is measured using dummy variables, with Democrat (reference category), Republican, Independent, and Other Party ID. Ideological identity is measured with values from 1 = Very Liberal to 5 = Very Conservative, which was included as a continuous measure.

Religious characteristics include measures of religious identity and religious commitment. Religious identity was measured with the following categories: Evangelical Protestant, Non-Evangelical Protestant, Catholic, Other Christian, Non-Christian Religion, Atheist, Agnostic, Nothing in Particular.^10^ We also include a religiosity index made from measures for religious service attendance (1 = Never to 6 = More than Once a Week), prayer frequency (1 = Never to 7 = Several Times a Day), and religious importance (1 = Not at All Important to 4 = Very Important). These measures were transformed into Z-scores and added together (alpha = 0.839). Table 1 presents unweighted and weighted descriptive statistics for all variables used in the analyses, both for the full sample and across racial groups separately. (The exact wording of all demographic variables is presented in Supplementary Material table S7.) All continuous variables were rescaled to range from 0 to 1 to make interpretation more intuitive.

**Table 1.** Descriptive statistics.

	Full sample				White				Black				Hispanic			
	Unweighted		Weighted		Unweighted		Weighted		Unweighted		Weighted		Unweighted		Weighted	
Variables	Mean	SD	Mean	SD	Mean	SD	Mean	SD	Mean	SD	Mean	SD	Mean	SD	Mean	SD
Self-identify as “Woke”	0.31	0.36	0.29	0.35	0.28	0.35	0.25	0.34	0.47	0.38	0.49	0.38	0.32	0.34	0.32	0.34
Self-identify as “Progressive”	0.53	0.39	0.51	0.39	0.53	0.39	0.49	0.39	0.57	0.36	0.58	0.35	0.56	0.39	0.57	0.38
Christian nationalism	0.35	0.30	0.37	0.30	0.34	0.31	0.37	0.32	0.42	0.27	0.44	0.27	0.38	0.29	0.36	0.29
Age	0.44	0.20	0.41	0.22	0.46	0.20	0.44	0.22	0.43	0.20	0.39	0.21	0.38	0.20	0.34	0.20
Education	0.50	0.30	0.48	0.30	0.51	0.30	0.51	0.30	0.41	0.27	0.42	0.27	0.44	0.29	0.38	0.27
Conservative ideology	0.50	0.29	0.52	0.29	0.51	0.30	0.54	0.30	0.41	0.24	0.42	0.24	0.49	0.28	0.47	0.27
Religiosity index	0.44	0.33	0.46	0.33	0.42	0.33	0.45	0.34	0.54	0.30	0.56	0.30	0.46	0.31	0.45	0.30


	%		%		%		%		%		%		%		%	
White	67.4		65.4													
Black	12.3		11.5													
Hispanic	11.9		13.8													
Other race	8.4		9.0													
Man	46.9		48.9		49.0		50.0		47.7		45.2		35.5		46.5	
Woman	51.9		49.9		50.0		49.0		51.4		53.9		64.5		53.5	
Nonbinary	1.2		1.1		1.0		1.0		0.9		0.9		0.0		0.0	
Income: Less than $30K	24.7		24.8		21.6		21.6		37.2		35.1		33.2		33.6	
Income: Between $30-60K	25.6		25.8		25.0		25.3		30.7		31.2		26.5		27.2	
Income: Between $60-100K	20.5		19.9		22.4		21.5		14.7		15.6		18.0		18.8	
Income: Between $100-200K	16.0		15.3		18.4		17.9		7.3		7.1		11.4		10.0	
Income: $200K or more	2.4		2.3		2.5		2.6		0.05		0.5		1.9		0.7	
Income: Did not say	10.8		11.9		10.1		11.1		9.6		10.6		9.0		9.6	
Southern residence	35.0		37.9		32.9		36.7		50.5		55.0		36.0		38.2	
Democrat	38.1		34.2		33.3		28.3		67.0		63.4		45.0		44.6	
Republican	24.1		27.4		28.2		33.7		5.0		6.1		21.8		18.7	
Independent	29.7		30.5		31.2		30.9		21.1		23.5		25.6		27.6	
Other party	8.0		8.0		7.4		7.2		6.9		7.1		7.6		9.0	
Evangelical Protestant	18.9		19.6		18.7		20.7		26.1		25.3		12.8		9.5	
Non-Evangelical Protestant	12.3		12.0		13.5		13.7		15.1		14.6		5.2		4.9	
Catholic	18.0		17.9		18.4		17.7		5.5		6.1		33.2		31.8	
Other Christian	1.6		1.5		2.1		2.0		0.5		0.4		0.5		0.2	
Non-Christian religion	11.2		11.6		9.1		8.7		17.0		19.5		9.5		13.2	
Atheist	7.1		6.5		8.5		8.0		2.8		2.2		4.7		4.2	
Agnostic	7.1		7.3		7.6		7.2		5.0		5.4		6.2		6.9	
Nothing in particular	23.7		23.6		22.1		22.0		28.0		26.4		28.0		29.4	
N	1,770				1,193				218				211			

*Source:* National Addiction and Social Attitudes Survey (Waves 1 and 3).

*Note:* Continuous variables are all rescaled to range from 0 to 1 for analyses.

### Plan of Analysis

The analysis proceeds as follows. Table 2 presents bivariate correlations between our Christian nationalism measure and the outcome measures for the full sample and across White, Black, and Hispanic Americans. Table 3 presents OLS regression models predicting self-identification with “woke” and “progressive.” Models 1 and 3 present the main effects models. Models 2 and 4 introduce interaction terms for Christian nationalism with Black, Hispanic, and Other Race Americans. In order to further assess racial differences in the association between Christian nationalism and self-identification with progressive identities, table 4 estimates OLS models for White, Black, and Hispanic Americans separately. Finally, table 5 follows recent research emphasizing the importance of partisan identity (see Djupe, Lewis, and Sokhey 2023) by running separate interaction models for Democrats, Republicans, and Independents. All models present unstandardized betas and *p* values calculated with robust standard errors to account for the sampling strategy and weights.

**Table 2.** Bivariate correlations between Christian nationalism and progressive identities for the full sample and across racial identities.

	Full sample		White		Black		Hispanic	
	*R*	*p* value	*r*	*p* value	*R*	*p* value	*r*	*p* value
Self-identify as “Woke”	−0.179	0.000	−0.274	0.000	0.178	0.008	−0.075	0.279
N	1,771		1,194		220		211	
Self-identify as “Progressive”	−0.355	0.000	−0.441	0.000	0.085	0.210	−0.121	0.079
N	1,770		1,193		218		211	

*Source:* National Addiction and Social Attitudes Survey (Waves 1 and 3).

*Note:* Analyses are weighted.

**Table 3.** Ordinary least squares regression models predicting self-identification with progressive identities.

	“Woke”				“Progressive”			
	Model 1		Model 2		Model 3		Model 4	
Variables	b	*p* value	b	*p* value	b	*p* value	b	*p* value
Christian nationalism	0.078	0.062	0.011	0.806	−0.025	0.606	−0.103	0.036
Black	0.132	0.000	0.002	0.969	−0.009	0.758	−0.147	0.004
Hispanic	0.013	0.663	−0.045	0.318	0.033	0.299	−0.071	0.139
Other race	−0.008	0.781	−0.010	0.838	−0.012	0.662	0.047	0.206
Age	−0.175	0.000	−0.171	0.000	−0.007	0.875	−0.003	0.947
Man	0.112	0.000	0.112	0.000	0.066	0.000	0.066	0.000
Nonbinary	0.066	0.416	0.068	0.401	0.022	0.768	0.027	0.705
Education	0.083	0.005	0.082	0.006	0.056	0.079	0.054	0.086
Income: Between $30–60K	0.001	0.978	−0.001	0.962	0.033	0.205	0.030	0.237
Income: Between $60–100K	−0.042	0.086	−0.048	0.055	−0.007	0.799	−0.013	0.611
Income: Between $100–200K	−0.033	0.217	−0.041	0.132	0.026	0.363	0.017	0.541
Income: $200K or more	−0.084	0.051	−0.092	0.036	−0.098	0.032	−0.104	0.024
Income: Did not say	0.018	0.553	0.010	0.733	−0.015	0.662	−0.022	0.515
Southern residence	0.049	0.005	0.047	0.006	0.013	0.466	0.010	0.564
Republican	−0.069	0.021	−0.058	0.056	−0.113	0.000	−0.102	0.001
Independent	−0.067	0.006	−0.063	0.011	−0.106	0.000	−0.101	0.000
Other party	−0.028	0.434	−0.016	0.641	−0.102	0.001	−0.090	0.003
Conservative ideology	−0.384	0.000	−0.374	0.000	−0.553	0.000	−0.537	0.000
Non-Evangelical Protestant	0.017	0.568	0.017	0.556	−0.024	0.435	−0.026	0.402
Catholic	0.007	0.787	0.008	0.757	0.015	0.620	0.014	0.644
Other Christian	0.056	0.288	0.062	0.230	0.057	0.335	0.066	0.268
Non-Christian religion	0.033	0.318	0.022	0.493	0.060	0.086	0.042	0.209
Atheist	0.090	0.056	0.082	0.079	0.116	0.021	0.101	0.041
Agnostic	−0.007	0.849	−0.007	0.855	0.049	0.269	0.044	0.316
Nothing in particular	0.041	0.178	0.041	0.174	0.026	0.438	0.022	0.505
Religiosity index	−0.011	0.782	−0.005	0.895	−0.050	0.223	−0.044	0.266
CN × Black			0.314	0.005			0.337	0.001
CN × Hispanic			0.165	0.118			0.296	0.010
CN × Other race			0.007	0.938			−0.168	0.037
Intercept	0.438	0.000	0.453	0.000	0.800	0.000	0.821	0.000
Adjusted R^2^	0.247		0.253		0.346		0.358	
N	1,771				1,770			

*Source:* National Addiction and Social Attitudes Survey (Waves 1 and 3).

*Note:* Analyses are weighted. Columns present unstandardized betas, and *p* values are estimated with robust standard errors. Excluded categories are White, Men, Income: Less than $30K, Republican, and Evangelical Protestant.

**Table 4.** Ordinary least squares regression models predicting self-identification with progressive identities across racial identity.

	“Woke”					
	White		Black		Hispanic	
Variables	b	*p* value	b	*p* value	b	*p* value
Christian nationalism	0.020	0.665	0.353	0.005	0.243	0.080
Age	−0.180	0.000	0.050	0.743	−0.405	0.005
Man	0.103	0.000	0.165	0.003	0.116	0.026
Nonbinary	0.011	0.927	0.404	0.088		
Education	0.092	0.008	0.090	0.374	−0.005	0.957
Income: Between $30–60K	−0.012	0.674	0.026	0.719	0.125	0.078
Income: Between $60–100K	−0.030	0.318	−0.078	0.357	−0.038	0.558
Income: Between $100–200K	−0.017	0.606	−0.101	0.385	0.014	0.840
Income: $200K or more	−0.094	0.076	−0.374	0.006	0.197	0.369
Income: Did not say	0.012	0.734	0.051	0.556	0.028	0.803
Southern residence	0.040	0.044	−0.022	0.682	0.094	0.093
Republican	−0.110	0.003	0.073	0.526	−0.102	0.247
Independent	−0.101	0.001	0.176	0.026	−0.115	0.078
Other party	−0.068	0.138	0.076	0.525	−0.002	0.981
Conservative ideology	−0.370	0.000	−0.395	0.001	−0.247	0.036
Non-Evangelical Protestant	0.023	0.497	−0.079	0.330	0.064	0.494
Catholic	−0.006	0.819	−0.068	0.484	0.102	0.141
Other Christian	0.049	0.407	0.179	0.193	−0.036	0.875
Non-Christian religion	0.044	0.276	−0.004	0.961	−0.104	0.195
Atheist	0.091	0.085	−0.344	0.044	0.083	0.493
Agnostic	−0.034	0.432	−0.233	0.146	0.170	0.188
Nothing in particular	0.017	0.630	−0.006	0.945	0.164	0.060
Religiosity index	0.026	0.552	−0.259	0.034	−0.030	0.813
Intercept	0.475	0.000	0.491	0.002	0.339	0.019
Adjusted R^2^	0.268		0.217		0.259	
N	1,194		218		211	


	“Progressive”					
	White		Black		Hispanic	
Variables	b	*p* value	b	*p* value	b	*p* value
Christian nationalism	−0.132	0.018	0.279	0.017	0.319	0.036
Age	0.058	0.291	0.205	0.102	−0.173	0.258
Man	0.081	0.000	0.030	0.549	0.073	0.177
Nonbinary	0.090	0.271	−0.130	0.713		
Education	0.052	0.160	0.028	0.776	0.003	0.981
Income: Between $30–60K	−0.019	0.547	0.149	0.020	0.174	0.021
Income: Between $60–100K	−0.044	0.165	−0.068	0.343	0.178	0.021
Income: Between $100–200K	−0.015	0.661	0.205	0.026	0.087	0.362
Income: $200K or more	−0.119	0.039	−0.425	0.002	0.178	0.515
Income: Did not say	−0.079	0.075	0.047	0.579	0.140	0.115
Southern residence	0.014	0.492	−0.115	0.019	0.024	0.688
Republican	−0.089	0.022	−0.034	0.772	−0.232	0.016
Independent	−0.111	0.000	0.093	0.167	−0.099	0.163
Other party	−0.089	0.022	0.074	0.515	−0.095	0.232
Conservative ideology	−0.597	0.000	−0.234	0.032	−0.485	0.000
Non-Evangelical Protestant	−0.027	0.449	−0.047	0.548	−0.168	0.164
Catholic	−0.004	0.907	0.095	0.314	0.003	0.976
Other Christian	0.045	0.493	0.366	0.008	−0.249	0.384
Non-Christian religion	0.007	0.874	0.015	0.835	−0.004	0.971
Atheist	0.095	0.091	−0.190	0.274	0.212	0.179
Agnostic	0.050	0.330	−0.212	0.165	−0.014	0.924
Nothing in particular	0.027	0.500	−0.097	0.241	−0.047	0.674
Religiosity index	0.018	0.691	−0.367	0.001	−0.247	0.096
Intercept	0.837	0.000	0.666	0.000	0.798	0.000
Adjusted R^2^	0.410		0.176		0.292	
N	1,193		218		211	

*Source:* National Addiction and Social Attitudes Survey (Waves 1 and 3).

*Note:* Analyses are weighted. Columns present unstandardized betas, and *p* values are estimated with robust standard errors. Excluded categories are Men, Income: Less than $30K, Republican, and Evangelical Protestant.

**Table 5.** Ordinary least squares regression models predicting self-identification with progressive identities across party identification.

	“Woke”					
	Democrat		Republican		Independent	
Variables	b	*p* value	b	*p* value	b	*p* value
Christian nationalism	−0.010	0.918	0.156	0.021	−0.062	0.391
Black	−0.130	0.063	0.070	0.789	0.220	0.055
Hispanic	−0.079	0.209	−0.027	0.756	0.002	0.977
Other race	−0.106	0.143	0.035	0.719	−0.004	0.966
Age	−0.105	0.136	−0.206	0.005	−0.123	0.090
Man	0.124	0.000	0.089	0.000	0.113	0.000
Nonbinary	0.065	0.619	−0.129	0.013	0.067	0.604
Education	0.096	0.073	0.149	0.004	0.068	0.200
Income: Between $30–60K	0.004	0.924	−0.045	0.324	0.027	0.564
Income: Between $60–100K	−0.012	0.778	−0.107	0.007	−0.049	0.278
Income: Between $100–200K	0.009	0.848	−0.084	0.083	−0.101	0.027
Income: $200K or more	0.131	0.294	−0.226	0.000	−0.102	0.134
Income: Did not say	0.068	0.302	−0.096	0.037	0.074	0.155
Southern residence	0.048	0.121	0.026	0.348	0.039	0.204
Conservative ideology	−0.363	0.000	−0.194	0.044	−0.390	0.000
Non-Evangelical Protestant	−0.034	0.508	0.056	0.256	0.007	0.911
Catholic	0.006	0.907	0.024	0.466	−0.049	0.325
Other Christian	0.140	0.579	0.066	0.177	0.034	0.742
Non-Christian religion	−0.056	0.307	0.111	0.031	−0.005	0.940
Atheist	0.031	0.691	0.215	0.014	0.020	0.800
Agnostic	0.074	0.319	−0.084	0.168	−0.084	0.216
Nothing in particular	0.044	0.468	0.030	0.539	−0.004	0.943
Religiosity index	0.086	0.216	−0.159	0.025	0.017	0.799
CN × Black	0.433	0.004	0.328	0.498	0.094	0.688
CN × Hispanic	0.131	0.422	0.241	0.160	0.037	0.822
CN × Other race	0.562	0.100	−0.012	0.938	−0.082	0.630
Intercept	0.395	0.000	0.290	0.003	0.429	0.000
Adjusted R^2^	0.145		0.229		0.257	
N	677		427		526	


	“Progressive”					
	Democrat		Republican		Independent	
Variables	b	*p* value	b	*p* value	b	*p* value
Christian nationalism	−0.222	0.020	0.131	0.133	−0.138	0.109
Black	−0.269	0.000	−0.151	0.648	0.013	0.902
Hispanic	−0.070	0.250	−0.088	0.529	−0.003	0.971
Other race	0.037	0.413	0.060	0.787	0.002	0.982
Age	0.130	0.051	0.006	0.951	−0.033	0.681
Man	0.044	0.111	0.113	0.000	0.048	0.125
Nonbinary	−0.019	0.920	−0.327	0.000	0.128	0.152
Education	0.134	0.011	0.102	0.097	0.039	0.466
Income: Between $30–60K	0.082	0.031	−0.123	0.023	0.036	0.442
Income: Between $60–100K	0.046	0.277	−0.226	0.000	0.031	0.553
Income: Between $100–200K	0.109	0.007	−0.171	0.004	0.022	0.689
Income: $200K or more	0.111	0.223	−0.277	0.000	−0.087	0.273
Income: Did not say	−0.060	0.337	−0.213	0.000	0.138	0.017
Southern residence	0.037	0.173	−0.003	0.918	−0.020	0.518
Conservative ideology	−0.304	0.000	−0.232	0.031	−0.773	0.000
Non-Evangelical Protestant	0.013	0.812	−0.022	0.704	−0.094	0.115
Catholic	−0.006	0.921	0.024	0.628	0.062	0.242
Other Christian	0.245	0.007	0.022	0.766	0.092	0.440
Non-Christian religion	0.030	0.613	0.059	0.324	0.061	0.315
Atheist	0.074	0.318	0.331	0.060	0.067	0.392
Agnostic	0.090	0.174	−0.017	0.864	0.011	0.882
Nothing in particular	0.048	0.444	0.033	0.548	−0.042	0.461
Religiosity index	−0.013	0.846	−0.173	0.034	−0.034	0.590
CN × Black	0.025	0.000	0.029	0.326	0.013	0.250
CN × Hispanic	0.018	0.043	0.012	0.299	0.011	0.271
CN × Other race	0.021	0.100	−0.007	0.680	−0.010	0.120
Intercept	0.628	0.000	0.511	0.000	0.862	0.000
Adjusted R^2^	0.212		0.160		0.375	
N	677		427		525	

*Source:* National Addiction and Social Attitudes Survey (Waves 1 and 3).

*Note:* Analyses are weighted. Columns present unstandardized betas, and *p* values are estimated with robust standard errors. Excluded categories are White, Men, Income: Less than $30K, and Evangelical Protestant.

## Results

Zero-order correlations for the full sample in table 2 show that Christian nationalism overall is negatively associated with feeling accurately described with the labels “woke” (r = −0.179, *p* < 0.001) and “progressive” (r = −0.355, *p* < 0.001). However, splitting the sample by racial identity shows clear divergences in these associations. For white Americans, there is a consistent, negative association between Christian nationalism and identifying with “woke” (r = −0.274, *p* < 0.001) and “progressive” (r = −0.0441, *p* < 0.001). For Black Americans, however, the associations between Christian nationalism and identification with “woke” (r = 0.178, *p* = 0.008) and “progressive” (r = 0.085, *p* = 0.210) are signed in the positive direction, though the latter is nonsignificant. For Hispanic Americans, the associations between Christian nationalism and identifying with “woke” (r = −0.075, *p* = 0.279) and “progressive (r = −0.121, *p* = 0.079) are both negative but nonsignificant. Full models will show that this latter association clearly diverges from the pattern we see for White Americans.

Turning to OLS regression analyses in table 3, Model 1 shows that Christian nationalism is not significantly associated with self-identifying with the term “woke” (b = 0.078; *p* = 0.062). Bivariate findings from table 1, however, suggest that this nonsignificant finding is masking considerable racial variation. Model 2 confirms this. The interaction term for Christian nationalism and Black Americans (β = 0.314, *p* = 0.005) is positive and statistically significant, indicating that as Christian nationalism increases, Black Americans’ likelihood of identifying with “woke” diverges from White Americans. Models 3 and 4 show similar patterns regarding identification with the term “progressive,” but one that includes Hispanic Americans as well. In the initial model (Model 3), the association with Christian nationalism is nonsignificant (b = 0.025; *p* = 0.606). However, when interaction terms are included in Model 4, the terms for Christian nationalism and Black Americans (β = 0.337, *p* = 0.001) and Hispanic Americans (β = 0.296, *p* = 0.010) are both statistically significant and positive.

**Figure 1.**
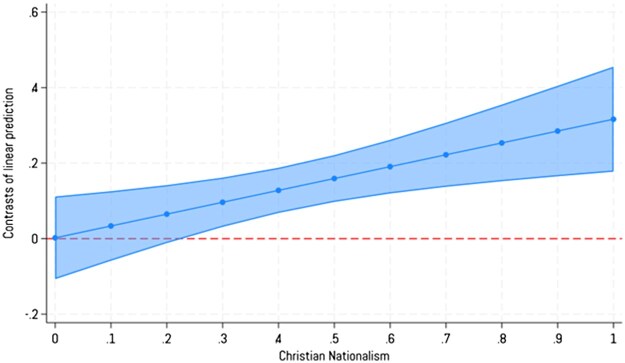
Predicted marginal contrast between White and Black Americans in identifying with “woke” across values of Christian nationalism. Error bands are 95 percent confidence intervals. The dotted line represents the effect for White Americans for contrast. Source: National Addiction and Social Attitudes Survey, Waves 1 and 3.

**Figure 2.**
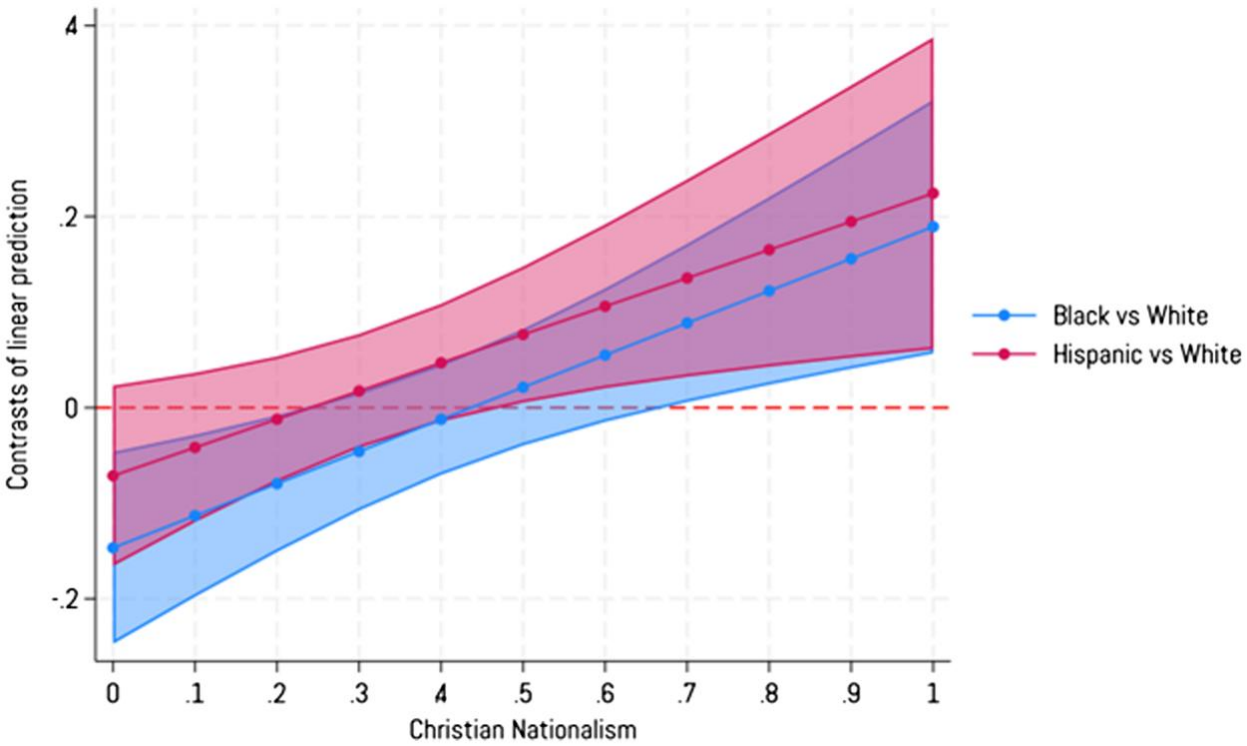
Predicted marginal contrast between White and Black or Hispanic Americans in identifying with “progressive” across values of Christian nationalism. Error bands are 95 percent confidence intervals. The dotted line represents the effect for White Americans for contrast. Source: National Addiction and Social Attitudes Survey, Waves 1 and 3.

Figures 1 and 2 show the marginal contrasts between racial groups to illustrate how the link between Christian nationalism and progressive identities diverges by racial identity. In figure 1 we see that even at lower values of Christian nationalism, Black Americans statistically diverge from White Americans (the dotted line at zero on the y-axis) in their likelihood to identify more with the term “woke.” Analyses in table 4 and graphing marginal probabilities in Supplementary Material figure S2 show that White Americans do not change across values of Christian nationalism, but the movement is wholly among Black Americans.

In figure 2, we show marginal contrasts for both Black and Hispanic Americans compared to White Americans in identifying with “progressive” as Christian nationalism increases. For Black Americans, those lowest on Christian nationalism are actually less likely than White Americans to identify with “progressive.” But as Christian nationalism increases, White Americans decline in their likelihood of identifying with “progressive” while Black Americans increase (see Supplementary Material figure S3: Panel A). We see a similar pattern for Hispanic Americans in figure 2. As Christian nationalism increases, Hispanic Americans statistically differ from White Americans in their likelihood of identifying with “progressive” around the midway point of the Christian nationalism scale. The trend lines for Hispanic and White Americans show divergence, as they do for Black Americans (see Supplementary Material figure S3: Panel B).^11^

By estimating models separately for our three racial groups of interest in table 4, we can have greater confidence that the link between Christian nationalism and identification with “woke” or “progressive” across racial groups is not an artifact, but truly reflects divergent patterns of association. Despite the significant bivariate association for White Americans (table 1), when we look at White Americans separately in table 4, we see that Christian nationalism is not significantly associated with identifying as “woke.” Ancillary analyses indicate that this is due to the inclusion of political controls (both partisan and ideological identity), suggesting the link between Christian nationalism and rejection of the term “woke” is fundamentally partisan for White Americans (Djupe, Lewis, and Sokhey 2023).

In contrast, the association between Christian nationalism and identifying with “woke” is positive and significant for Black Americans (b = 0.353, *p* = 0.005). And though the association for Hispanic Americans is signed in the positive direction, it does not quite attain statistical significance at the 0.05 level (b = 0.243, *p* = 0.080). Similarly, the finding regarding identification with “progressive” is largely identical to what we see in the interaction terms and marginal probabilities. Christian nationalism is significantly, negatively associated with this self-identification with “progressive” for White Americans (b = −0.132, *p* = 0.018) even as it is significant and positive for Black (b = 0.279, *p* = 0.017) and Hispanic Americans (b = 0.319, *p* = 0.036).

Finally, recent studies (Djupe, Lewis, and Sokhey 2023; Perry 2023) have made the case that Christian nationalism must also be understood within the context of American partisanship and thus we reproduce our key interaction models from table 3 in table 5 with samples split by party identification. The interaction terms for Christian nationalism and racial identity are only statistically significant for those in the Democratic Party. However, this different pattern may simply be due to the fact that such a large proportion of Black and Hispanic Americans are Democrats rather than Republicans or Independents. Moreover, among Republicans and Independents, the interaction terms are still signed in the same direction as for Democrats. And marginal contrasts presented in Supplementary Material figures S4–S6 affirm the patterns even if they do not attain statistical significance. Thus, these results do not suggest meaningfully divergent patterns across party identification.

## Discussion and Conclusions

Though discussions of Christian nationalism generally associate the construct with partisan and ideological conservatism in opposition to all things progressive, a burgeoning research has shown that racial identity can often moderate the link between Christian nationalist views and progressive stances on political issues and partisan leanings (Perry and Whitehead 2019; Armaly, Buckley, and Enders 2022; Gorski and Perry 2022; McDaniel, Nooruddin, and Shortle 2022; Perry et al. 2022, 2024a, 2024b; Perry and Schleifer 2023). Extending this idea to the example of progressive social identities, our findings largely affirm our expectations. Using a composite Christian nationalism scale that incorporates previously tested measures of ideology and a measure of self-identification, we find that Christian nationalism is either unassociated or negatively associated with identifying with progressive identities among White Americans. However, the more Black Americans affirm Christian nationalism, the more likely they are to identify with “woke,” and both Black and Hispanic Americans become more likely to identify with “progressive.”

These results extend our understanding of both Christian nationalism and progressive social identities in a number of important ways. First, though we are generally in agreement with Djupe, Lewis, and Sokhey’s (2023, p. 27) observation that Christian nationalism often functions as “a Republican project,” this clearly must be contextualized by social factors, most notably racial and ethnic identity. As previous research on race, religion, and politics would lead us to expect, religious language is interpreted through the lens of different theological traditions and ethno-racial group interest (Thurman 1976; McDaniel and Ellison 2008; McKenzie and Rouse 2013; Wilde and Glassman 2016; Phillips 2018; Perry 2023; Al-Kire et al. 2024). Indeed, recent research on Christian nationalism strongly supports this idea (Perry and Whitehead 2019; Armaly, Buckley, and Enders 2022; Gorski and Perry 2022; McDaniel, Nooruddin, and Shortle 2022; Perry et al. 2022, 2024a, 2024b; Perry and Schleifer 2023). Our work documents that, among Black and Hispanic Americans, embracing Christian nationalist views and identity is consistent with (and indeed conductive to) identifying with terms like “woke” or “progressive.” As Djupe, Lewis, and Sokhey (2023) rightly suggest in their analysis, partisan identity is so central to analyzing the link between religion and politics that studies must consider how associations vary across partisan identity. We would affirm the same methodological rule for racial identity, consistent with recommendations from sociologists of religion (e.g., Wilde and Glassman 2016; Edgell and Yukich 2020; Perry 2023, 2024).

Related to this, our research not only extends our understanding of Christian nationalism, but helps us understand how progressive political identities are differentially shaped by religious and racial beliefs and identities. Being “woke” or “progressive,” for example, does not necessarily imply secularity, but in fact seems to be endorsed by Americans of color who recognize a tighter historical relationship between Christianity in the United States and would support such a relationship in the future. As others have recognized (Gorski and Perry 2022; McDaniel, Nooruddin, and Shortle 2022), this reflects the aspirational, liberating, social-justice-oriented theology of such communities that centers their own experiences. This reality also reflects the challenge that progressive coalitions face in the United States. Unlike Americans on the right who are increasingly unified in partisan, ideological, religious, and racial identities (Mason 2018; Perry 2023), Americans on the left must not only work through challenges associated with greater racial diversity, but include groups of Americans who are dogmatically opposed to Christian nationalist rhetoric and beliefs and people of color who often enthusiastically endorse them as part of their own “Sacred Cosmos” (Whitehead and Perry 2020).

One apparent inconsistency that actually supports our argument about Christian nationalism sacralizing and supporting the status of “our people” was the fact that, though Christian nationalism was positively associated with self-describing as “progressive” for both Black and Hispanic Americans, identification with “woke” was only significant for Black Americans (table 4; Supplementary Material figure S2). Though this difference is not particularly substantial, given that the association was marginally significant for Hispanic Americans (table 4), we propose that the difference may be attributed to the historical connection between the term “woke” and the Black community, thus making the term more racially coded as “Black” and thus foregrounding the in-group connection for Black Americans. In addition to this, there is also the possibility that the category “Hispanic” in the survey captures a wider spectrum of ethnic identities and salience that may mitigate identification with the term.

Several data limitations should be acknowledged in order to chart a path for future research. First, the progressive identities in question are limited, and future studies could make use of a variety of identities and labels to see how Christian nationalist ideology corresponds to labels like “liberal,” “socialist,” “passivist,” “leftist,” or “left-wing.” Conversely, it would also be interesting to examine whether Christian nationalism makes Black and Hispanic Americans less inclined to embrace social identities more associated with political conservatism, such as “nativist,” “capitalist,” “nationalist,” “pro-military,” or “right-wing.” Second, though the causal ordering theorized in this study seems the most plausible, the analysis is nonetheless cross-sectional, and experimental studies would be useful in disentangling some of the relationships. For example, studies could consider whether priming Christian nationalist views (perhaps with vignettes about Christianity’s unique influence on American law and culture) among a diverse sample of participants inclines Americans to identify with different political identities and whether that treatment effect varies by racial identity.

Although we demonstrate that our Christian nationalism measure is both robust to different measurement options (see Supplementary Material tables S5 and S6) and hangs together well across racial identities, another unfortunate limitation is that we are not able to compare it with other common Christian nationalism scales (e.g., Whitehead and Perry 2020; Djupe, Lewis, and Sokhey 2023). Future studies should continue to test for the best ways to measure the construct, which will inevitably involve a larger battery of questions that have been used in recent years to tease out different dimensions of the construct.

A final data limitation worth addressing is the notable absence of attention to Asian Americans, due to limited sample size. Research on how Asian Americans connect Christian nationalist views to politics is woefully sparse, largely for this reason. Future studies would ideally oversample for Asian Americans to include their perspectives as well. Moreover, for both Asian and Hispanic respondents, additional questions about foreign-born versus native-born status might also be helpful. And to the extent that sample sizes allow, questions about national heritage could also be meaningful. For example, Asian Americans from South Korea might have a different relationship to religion and politics than Vietnamese or Chinese Americans do, and we might see similar differences for Hispanic Americans from Puerto Rico, Spain, and Cuba.

Data limitations notwithstanding, the current study offers important insights to further our understanding of religion, race, and politics. Though scholars, pundits, and journalists are increasingly discussing the threat of reactionary and antidemocratic *White* Christian nationalism, the whole premise of research in that area is that Christian nationalist views do not operate the same way across diverse sets of Americans. The same views that seem to correspond with a desire to preserve racial, religious, and gender hierarchies in society may support more progressive views and identities among those Americans who have experienced marginalization and for whom religion has served as a liberating and empowering resource (McDaniel and Ellison 2008; McKenzie and Rouse 2013; Phillips 2018).

## Supplementary Material

nfaf009_Supplementary_Data

## Data Availability

Replication data and documentation are available at https://doi.org/10.7910/DVN/DP1CEL.

## Footnotes

1 Martin Luther King Jr. provided several clear examples of this. In his 1958 sermon “Paul’s Letter to American Christians,” he decries capitalism’s exploitation of the poor: “Oh, America, how often you have taken necessities from the masses to give luxuries to the classes? If you are to be a truly Christian nation, you must solve this problem.” Elsewhere, King (1963) reminded Birmingham clergy that “[Black Americans] will win our freedom because the sacred heritage of our nation and the eternal will of God are embodied in our echoing demands.” Later in his letter, King declared that those who fight for civil rights are fighting for “what is best in the American dream and for the most sacred values in our Judeo-Christian heritage…”

2 The NASAS data are currently proprietary, but will be posted at www.TheARDA.com in the future, and the data and code for this study are available at the lead author’s Harvard dataverse for replication purposes.

3 The outcome variables and our specific items for our Christian nationalism measure were only asked in Wave 3 of the NASAS and not in Wave 1. Wave 2 included follow-up questions related to addictive behaviors related to gambling and sex. It did not contain any attitudinal questions used in Wave 3.

4 Due to nonresponse, the use of online nonprobability surveys has increased in recent years and certain vendors are better than others (Kennedy et al. 2016). A recent study comparing results from YouGov’s opt-in matching samples to those of probability-based samples like the General Social Survey found they compare favorably (Graham, Pickett, and Cullen 2021).

5 See Supplementary Material table S2, where we present the percentage distribution of responses to the identity-based questions used in this study. In supplementary analyses, when we ran models omitting the “Undecided” category instead of using them as a middle category, the results are substantively identical (see Supplementary Material table S4).

6 See Supplementary Material table S3, where we present the percentage distribution of responses to our ideological questions used in constructing the Christian nationalism scale.

7 We also tested models using a Christian nationalism scale with only the first four items (alpha = 0.905), and results were substantively identical (see Supplementary Material table S5).

8 Examining Cronbach’s alpha scores for this measure across racial groups showed generally consistent results (White = 0.921; Black = 0.846; Hispanic = 0.894). Principal components factor analyses also revealed that these items loaded cleanly onto a single factor, with loadings ranging between 0.799 and 0.901 and explaining 73.6 percent of the variance. Principal components factor analyses by racial group again showed that a single factor best explained the relationship (White, loadings = 0.818 to 0.913; 76.1 percent explained; Black, loadings = 0.742 to 0.841; 62.6 percent explained; Hispanic, loadings 0.728 to 0.895; 70.9 percent explained).

9 The survey also included a statement similar to one used in previous indexes of Christian nationalism: “It is critical that our government maintain a separation of church and state.” However, as with other recent studies (e.g., Djupe, Lewis, and Sokhey 2023), we found that this measure was not correlated strongly with the other measures (coefficients range from 0.31 to 0.39) and did not load as cleanly onto our index, reducing the alpha for the full sample and across all racial groups. Thus, we left this out of the analysis. The results were substantively identical with the measure included (see Supplementary Material table S6).

10 Evangelical and Non-Evangelical Protestants are measured by taking respondents who self-identified as “Protestant” and then dividing them up into Protestants who self-identified as born-again or Evangelical Christians (Evangelical Protestants) and those who did not (Non-Evangelical Protestants).

11 As a robustness check, in supplementary analyses (not shown) we ran all models from table 3 for each item in our Christian nationalism index separately. The patterns we identify in figures 1 and 2 were substantively identical for each item (unsurprising given how strongly related they are), and thus we are more confident that our findings are not due to isolated associations with particular items.
