# Supplementary material for: White or Woke Christian Nationalists? How Race Moderates the Link Between Christian Nationalism and Progressive Identities
Source: Public Opin Q. 2025 May 13;89(1):98–124. doi: 10.1093/poq/nfaf009 (PMC12166974; doi:10.1093/poq/nfaf009)

SUPPLEMENTARY MATERIALS

White or Woke Christian Nationalists? How Race Moderates the Link Between Christian Nationalism and Progressive Identities

Samuel L. Perry
Department of Sociology
University of Oklahoma
samperry@ou.edu

Allyson F. Shortle
Department of Political Science
University of Oklahoma
allysonshortle@ou.edu

Eric L. McDaniel
Department of Political Science
University of Texas at Austin
emcdaniel@austin.utexas.edu

Joshua B. Grubbs
Department of Psychology
University of New Mexico
joshuagrubbs12@unm.edu

TABLE OF CONTENTS

1. **Table S1:** Comparison of NASAS Original Sample, Analytic Sample, and 2018 GSS on Key Demographic Variables.
2. **Table S2:** Distribution of Identification Measures
3. **Table S3:** Distribution of Christian Nationalism Ideology Measures
4. **Table S4:** Ordinary least squares regression models predicting self-identification with progressive identities using outcome variables without the "Undecided" category.
5. **Table S5:** Ordinary least squares regression models predicting self-identification with progressive identities using the Christian Nationalism index without the question asking how well the term "Christian nationalist" describes the respondent.
6. **Table S6:** Ordinary least squares regression models predicting self-identification with progressive identities using the Christian Nationalism index with the question about Church-State separation.
7. **Table S7:** Question Wording for All Measures Used in the Analyses.
8. **Figure S1:** Screenshots of outcome questions and Christian nationalism questions as they appear in the National Addiction and Social Attitude Survey, Wave 3.
9. **Figure S2:** Predicted marginal probabilities of identifying with "woke" for White and Black Americans across values of Christian nationalism. Error bars are 95% confidence intervals. Source: National Addiction and Social Attitudes Survey, Waves 1 and 3.
10. **Figure S3:** Predicted marginal probabilities of identifying with "progressive" for White and Black or Hispanic Americans across values of Christian nationalism. Error bars are 95% confidence intervals. Source: National Addiction and Social Attitudes Survey, Waves 1 and 3.
11. **Figure S4:** Marginal Contrasts of Christian nationalism and being Black vs. White on identifying as "woke" among Democrats, Republicans, and Independents. Error bars are 95% confidence intervals. The dotted red line represents the effect for White Americans for contrast. Source: National Addiction and Social Attitudes Survey, Waves 1 and 3.
12. **Figure S5:** Marginal Contrasts of Christian nationalism and being Black vs. White on identifying as "progressive" among Democrats, Republicans, and Independents. Error bars are 95% confidence intervals. The dotted red line represents the effect for White Americans for contrast. Source: National Addiction and Social Attitudes Survey, Waves 1 and 3.
13. **Figure S6:** Marginal Contrasts of Christian nationalism and being Hispanic vs. White on identifying as "progressive" among Democrats, Republicans, and Independents. Error bars are 95% confidence intervals. The dotted red line represents the effect for White Americans for contrast. Source: National Addiction and Social Attitudes Survey, Waves 1 and 3.

Table S1: Comparison of NASAS Original Sample, Analytic Sample, and 2018 GSS on Key Demographic Variables.

	NASAS W1 (2022) Sample			NASAS Analytic Sample			2018 GSS		
	Range	Mean or %	SD	Range	Mean or %	SD	Range	Mean or %	SD
Male	0-1	49%		0-1	49%		0-1	46%	
Woman	0-1	50%		0-1	50%				
Non-binary	0-1	1%		0-1	1%				
White	0-1	63%		0-1	66%		0-1	72%	
Black	0-1	12%		0-1	12%		0-1	15%	
Latino	0-1	16%		0-1	14%				
Other Race	0-1	9%		0-1	9%		0-1	13%	
Age	20-97	49.9	17.3	20-97*	51.8	16.8	18-89	46.6	17.7
Married	0-1	41%		0-1	43%		0-1	49%	
BA Degree or Higher	0-1	31%		0-1	31%		0-1	31%	
Southern US	0-1	38%		0-1	38%		0-1	40%	
Full-time Employed	0-1	35%		0-1	35%		0-1	50%	
Republican	0-1	26%		0-1	27%		0-1	35%	
Democrat	0-1	35%		0-1	34%		0-1	43%	
Independent	0-1	30%		0-1	30%		0-1		
Other	0-1	10%		0-1	8%		0-1	22%	
Born-Again/Evangelical Prot.	0-1	18%		0-1	20%		0-1	23%	

Note: Because Wave 3 of the NASAS was fielded within 6-7 months of Wave 1, the sample still contained respondents who were age 20 and the 2 respondents who were 97 in Wave 1 were still 97 by Wave 3. Numbers are from weighted data.

Table S2: Distribution of Identification Measures

Response Options	Woke	Progressive	Christian Nationalist
Not at all	49.04	23.63	57.47
Not very well	11.95	16.30	11.00
Undecided	15.16	9.42	8.57
Somewhat well	14.21	24.03	13.25
Very well	9.64	26.62	9.70
N	1,774	1,774	1,173

Source NASAS Wave 3 (unweighted).

Table S3: Distribution of Christian Nationalism Ideology Measures

Response Options	God's Plan	Christian Nation	Founding Docs	Truly American
Strongly disagree	32.86	47.86	33.30	41.30
Disagree	7.10	10.15	11.60	10.25
Neither agree nor disagree	32.64	23.62	27.15	22.93
Agree	14.99	8.57	14.70	12.68
Strongly agree	12.4	9.81	13.18	12.85
N	1,774	1,774	1,174	1,175

Source NASAS Wave 3 (unweighted).

Table S4: Ordinary least squares regression models predicting self-identification with progressive identities using outcome variables without the "Undecided" category.

	"Woke"				"Progressive"			
	Model 1		Model 2		Model 3		Model 4	
Variables	b	p value	b	p value	b	p value	b	p value
Christian nationalism	0.052	0.246	-0.023	0.596	-0.015	0.766	-0.097	0.055
Black	0.161	0.000	0.008	0.900	-0.010	0.761	-0.154	0.005
Hispanic	0.007	0.821	-0.049	0.300	0.031	0.364	-0.075	0.125
Other Race	-0.002	0.948	-0.020	0.698	-0.011	0.716	0.036	0.344
Age	-0.177	0.000	-0.173	0.000	-0.007	0.896	-0.003	0.946
Man	0.101	0.000	0.102	0.000	0.067	0.000	0.067	0.000
Non-Binary	0.095	0.292	0.098	0.281	0.018	0.822	0.023	0.758
Education	0.094	0.004	0.092	0.005	0.065	0.050	0.063	0.053
Income: Between $30-60K	0.015	0.598	0.015	0.583	0.042	0.128	0.039	0.151
Income: Between $60-100K	-0.028	0.297	-0.032	0.228	0.005	0.858	-0.001	0.984
Income: Between $100-200K	-0.014	0.617	-0.021	0.463	0.032	0.278	0.023	0.438
Income: $200K or more	-0.063	0.155	-0.071	0.117	-0.084	0.069	-0.089	0.057
Income: Did not say	0.012	0.723	0.005	0.883	-0.012	0.746	-0.020	0.590
Southern residence	0.048	0.010	0.047	0.011	0.011	0.555	0.009	0.631
Democrat	-0.057	0.078	-0.047	0.153	-0.112	0.001	-0.100	0.003
Independent	-0.066	0.013	-0.063	0.019	-0.113	0.000	-0.110	0.000
Other Party	-0.024	0.529	-0.011	0.784	-0.109	0.002	-0.092	0.007
Conservative Ideology	-0.424	0.000	-0.410	0.000	-0.575	0.000	-0.558	0.000
Non-Evangelical Protestant	0.012	0.702	0.012	0.704	-0.017	0.587	-0.017	0.593
Catholic	-0.015	0.540	-0.013	0.604	0.011	0.721	0.011	0.731
Other Christian	0.096	0.060	0.104	0.042	0.064	0.236	0.074	0.171
Non-Christian Religion	0.032	0.368	0.022	0.531	0.067	0.070	0.048	0.173
Atheist	0.076	0.135	0.069	0.167	0.126	0.013	0.109	0.029
Agnostic	-0.021	0.598	-0.021	0.610	0.052	0.233	0.048	0.269
Nothing in Particular	0.037	0.259	0.039	0.231	0.036	0.316	0.034	0.339
Religiosity Index	-0.002	0.965	0.005	0.915	-0.037	0.401	-0.035	0.412
CN × Black			0.378	0.005			0.358	0.001
CN × Hispanic			0.164	0.148			0.304	0.010
CN × Other Race			0.054	0.576			-0.132	0.111
Intercept	0.430	0.000	0.445	0.000	0.785	0.000	0.807	0.000
Adjusted R^2	0.275		0.283		0.373		0.385	
N	1,502				1,603			

Source: National Addiction and Social Attitudes Survey (Wave 1 and 3).

Note: Analyses are weighted. *p* values are estimated with robust standard errors. Excluded categories are White, Men, Income: Less than $30K, Republican, and Evangelical Protestant.

Table S5: Ordinary least squares regression models predicting self-identification with progressive identities using the Christian Nationalism index without the question asking how well the term "Christian nationalist" describes the respondent.

	"Woke"				"Progressive"			
	Model 1		Model 2		Model 3		Model 4	
Variables	b	p value	b	p value	b	p value	b	p value
Christian nationalism	-0.012	0.770	-0.066	0.109	-0.116	0.015	-0.183	0.000
Black	0.139	0.000	0.020	0.716	-0.003	0.923	-0.124	0.014
Hispanic	0.014	0.630	-0.041	0.374	0.034	0.289	-0.073	0.140
Other Race	-0.007	0.807	0.001	0.991	-0.011	0.688	0.046	0.214
Age	-0.167	0.000	-0.165	0.000	0.002	0.960	0.004	0.934
Man	0.113	0.000	0.113	0.000	0.067	0.000	0.067	0.000
Non-Binary	0.068	0.401	0.069	0.392	0.024	0.745	0.026	0.707
Education	0.071	0.017	0.069	0.021	0.042	0.182	0.039	0.211
Income: Between $30-60K	0.001	0.965	0.000	0.996	0.033	0.200	0.031	0.226
Income: Between $60-100K	-0.042	0.086	-0.046	0.064	-0.007	0.804	-0.012	0.662
Income: Between $100-200K	-0.036	0.184	-0.042	0.122	0.024	0.400	0.016	0.564
Income: $200K or more	-0.092	0.037	-0.096	0.030	-0.103	0.026	-0.106	0.022
Income: Did not say	0.019	0.527	0.013	0.663	-0.013	0.701	-0.019	0.582
Southern residence	0.050	0.004	0.048	0.005	0.014	0.447	0.011	0.512
Democrat	-0.058	0.052	-0.049	0.105	-0.102	0.002	-0.094	0.004
Independent	-0.065	0.008	-0.061	0.012	-0.102	0.000	-0.099	0.000
Other Party	-0.025	0.480	-0.016	0.655	-0.098	0.002	-0.090	0.003
Conservative Ideology	-0.360	0.000	-0.351	0.000	-0.528	0.000	-0.514	0.000
Non-Evangelical Protestant	0.009	0.743	0.011	0.707	-0.030	0.323	-0.031	0.309
Catholic	0.003	0.907	0.006	0.809	0.012	0.678	0.013	0.656
Other Christian	0.057	0.278	0.067	0.205	0.063	0.284	0.074	0.205
Non-Christian Religion	0.027	0.404	0.020	0.547	0.055	0.116	0.040	0.237
Atheist	0.076	0.110	0.069	0.142	0.100	0.047	0.086	0.084
Agnostic	-0.021	0.586	-0.021	0.603	0.035	0.422	0.030	0.483
Nothing in Particular	0.032	0.300	0.033	0.276	0.017	0.605	0.015	0.659
Religiosity Index	0.017	0.657	0.021	0.587	-0.022	0.598	-0.019	0.638
CN × Black			0.278	0.011			0.284	0.005
CN × Hispanic			0.147	0.130			0.286	0.012
CN × Other Race			-0.019	0.825			-0.151	0.045
Intercept	0.450	0.000	0.463	0.000	0.813	0.000	0.834	0.000
Adjusted R^2	0.245		0.250		0.350		0.360	
N	1,172				1,171			

Source: National Addiction and Social Attitudes Survey (Wave 1 and 3).
Note: Analyses are weighted. *p* values are estimated with robust standard errors. Excluded categories are White, Men, Income: Less than $30K, Republican, and Evangelical Protestant.

Table S6: Ordinary least squares regression models predicting self-identification with progressive identities using the Christian Nationalism index with the question about Church-State separation.

	"Woke"				"Progressive"			
	Model 1		Model 2		Model 3		Model 4	
Variables	b	p value	b	p value	b	p value	b	p value
Christian nationalism	0.118	0.022	0.036	0.503	0.042	0.485	-0.063	0.297
Black	0.132	0.000	-0.045	0.514	-0.014	0.645	-0.207	0.002
Hispanic	0.013	0.648	-0.070	0.240	0.032	0.303	-0.135	0.035
Other Race	-0.009	0.768	0.004	0.955	-0.013	0.642	0.080	0.115
Age	-0.180	0.000	-0.177	0.000	-0.013	0.772	-0.012	0.789
Man	0.112	0.000	0.112	0.000	0.065	0.000	0.066	0.000
Non-Binary	0.065	0.422	0.068	0.399	0.021	0.785	0.027	0.707
Education	0.085	0.004	0.084	0.005	0.064	0.044	0.061	0.050
Income: Between $30-60K	-0.000	0.997	-0.002	0.936	0.033	0.211	0.029	0.254
Income: Between $60-100K	-0.043	0.080	-0.048	0.052	-0.007	0.786	-0.015	0.573
Income: Between $100-200K	-0.034	0.203	-0.041	0.132	0.027	0.346	0.019	0.508
Income: $200K or more	-0.086	0.046	-0.093	0.033	-0.094	0.037	-0.100	0.028
Income: Did not say	0.016	0.590	0.009	0.752	-0.016	0.635	-0.024	0.482
Southern residence	0.049	0.005	0.047	0.006	0.013	0.478	0.010	0.583
Democrat	-0.071	0.017	-0.062	0.041	-0.120	0.000	-0.111	0.000
Independent	-0.067	0.006	-0.065	0.009	-0.107	0.000	-0.104	0.000
Other Party	-0.028	0.430	-0.017	0.630	-0.104	0.001	-0.092	0.002
Conservative Ideology	-0.384	0.000	-0.373	0.000	-0.567	0.000	-0.549	0.000
Non-Evangelical Protestant	0.018	0.540	0.019	0.514	-0.019	0.534	-0.021	0.493
Catholic	0.007	0.765	0.009	0.708	0.018	0.561	0.017	0.575
Other Christian	0.055	0.293	0.062	0.232	0.056	0.344	0.065	0.271
Non-Christian Religion	0.033	0.310	0.023	0.480	0.063	0.067	0.044	0.183
Atheist	0.091	0.053	0.085	0.070	0.125	0.013	0.110	0.027
Agnostic	-0.006	0.879	-0.006	0.874	0.057	0.195	0.051	0.245
Nothing in Particular	0.043	0.162	0.043	0.155	0.033	0.340	0.028	0.408
Religiosity Index	-0.015	0.699	-0.009	0.825	-0.069	0.090	-0.063	0.110
CN × Black			0.389	0.004			0.427	0.001
CN × Hispanic			0.200	0.136			0.405	0.004
CN × Other Race			-0.028	0.807			-0.220	0.039
Intercept	0.419	0.000	0.446	0.000	0.786	0.000	0.825	0.000
Adjusted R^2	0.248		0.255		0.346		0.360	
N	1,171				1,170			

Source: National Addiction and Social Attitudes Survey (Wave 1 and 3).

Note: Analyses are weighted. *p* values are estimated with robust standard errors. Excluded categories are White, Men, Income: Less than $30K, Republican, and Evangelical Protestant.

Table S7: Question Wording for All Measures Used in the Analyses.

Variable	Question wording	Response Options
Age	In what year were you born?	Open integer textbox
Education	What is the highest level of education you have completed?	1. No HS<br>2. High school graduate<br>3. Some college<br>4. 2-year<br>5. 4-year<br>6. Post-grad
Political Ideology	In general, how would you describe your own political viewpoint?	1. Very liberal<br>2. Liberal<br>3. Moderate<br>4. Conservative<br>5. Very conservative<br>6. Not Sure
Political Party	Generally speaking, do you see yourself as a…?	1. Democrat<br>2. Republican<br>3. Independent<br>4. Other<br>5. Not Sure
Religious Service Attendance	Aside from weddings and funerals, how often do you attend religious services?	1. More than once a week<br>2. Once a week<br>3. Once or twice a month<br>4. A few times a year<br>5. Seldom<br>6. Never<br>7. Don't know
Prayer Frequency	Outside of attending religious services, how often do you pray?	1. Several times a day<br>2. Once a day<br>3. A few times a week<br>4. Once a week<br>5. A few times a month<br>6. Seldom<br>7. Never<br>8. Don't know
Religious Importance	How important is religion in your life?	1. Very important<br>2. Somewhat important<br>3. Not too important<br>4. Not at all important
Race	What racial or ethnic group best describes you?	1. White<br>2. Black<br>3. Hispanic<br>4. Asian<br>5. Native American<br>6. Middle Eastern<br>7. Mixed<br>8. Other
Gender	What is your gender?	1. Man<br>2. Woman<br>3. Non-binary<br>4. Other
Household Income	Thinking back over the last year, what was your family's annual income?	1. Less than $10,000<br>2. $10,000-$19,999<br>3. $20,000-$29,999<br>4. $30,000-$39,999<br>5. $40,000-$49,999

6. $50,000-$59,999
7. $60,000-$69,999
8. $70,000-$79,999
9. $80,000-$99,999
10. $100,000-$119,999
11. $120,000-$149,999
12. $150,000-$199,999
13. $200,000-$249,999
14. $250,000-$349,999
15. $350,000-$499,999
16. $500,000 or more
97. Prefer not to say

State of Residence	What is your state of residence?	1 Alabama<br>2 Alaska<br>4 Arizona<br>5 Arkansas<br>6 California<br>8 Colorado<br>9 Connecticut<br>10 Delaware<br>11 District of Columbia<br>12 Florida<br>13 Georgia<br>15 Hawaii<br>16 Idaho<br>17 Illinois<br>18 Indiana<br>19 Iowa<br>20 Kansas<br>21 Kentucky<br>22 Louisiana<br>23 Maine<br>24 Maryland<br>25 Massachusetts<br>26 Michigan<br>27 Minnesota<br>28 Mississippi<br>29 Missouri<br>30 Montana<br>31 Nebraska<br>32 Nevada<br>33 New Hampshire<br>34 New Jersey<br>35 New Mexico<br>36 New York<br>37 North Carolina<br>38 North Dakota<br>39 Ohio<br>40 Oklahoma<br>41 Oregon<br>42 Pennsylvania<br>44 Rhode Island<br>45 South Carolina<br>46 South Dakota<br>47 Tennessee<br>48 Texas

		49 Utah<br>50 Vermont<br>51 Virginia<br>53 Washington<br>54 West Virginia<br>55 Wisconsin<br>56 Wyoming
Religious Tradition	What is your present religion, if any?	1. Protestant<br>2. Roman Catholic<br>3. Mormon<br>4. Eastern or Greek Orthodox<br>5. Jewish<br>6. Muslim<br>7. Buddhist<br>8. Hindu<br>9. Atheist<br>10. Agnostic<br>11. Nothing in particular<br>12. Something else
Born-Again Protestant	Would you describe yourself as a born again or evangelical Christian?	1. Yes<br>2. No

Figure S1: Screenshots of outcome questions and Christian nationalism questions as they appear in the National Addiction and Social Attitude Survey, Wave 3.

A. Identity Outcome Questions

YouGov

How well do the following words describe you?

	Not at all	Not very well	Somewhat well	Very well	Undecided
Pro-choice	○	○	○	○	○
Patriotic	○	○	○	○	○
Pro-life	○	○	○	○	○
Progressive	○	○	○	○	○
Christian nationalist	○	○	○	○	○
Woke	○	○	○	○	○

B. Christian Nationalism Questions (not including the question about identifying with the term “Christian nationalist.”

YouGov

Please indicate your level of agreement with the following statements

	Strongly disagree	Disagree	Neither agree nor disagree	Agree	Strongly agree
I consider founding documents like the Declaration of Independence and the US Constitution to be divinely inspired.	○	○	○	○	○
The percentage of Americans who share my views on the role of religion in government is growing.	○	○	○	○	○
I would support state governments arresting women who have abortions.	○	○	○	○	○
I would support legally nullifying all current same-sex marriages.	○	○	○	○	○
I consider being a Christian an important aspect of being truly American.	○	○	○	○	○
It is critical that our government maintains a separation of church and state.	○	○	○	○	○
I would support the Supreme Court overturning their 2015 decision that legalized same-sex marriages.	○	○	○	○	○
I support the 2022 Supreme Court decision that overturned Roe v. Wade.	○	○	○	○	○
The federal government should declare the United States a Christian nation.	○	○	○	○	○
America holds a special place in God's plan.	○	○	○	○	○
My views on the role of religion in government represent the views of most Americans.	○	○	○	○	○

Figure S2: Predicted marginal probabilities of identifying with “woke” for White and Black Americans across values of Christian nationalism. Error bars are 95% confidence intervals. Source: National Addiction and Social Attitudes Survey, Waves 1 and 3.

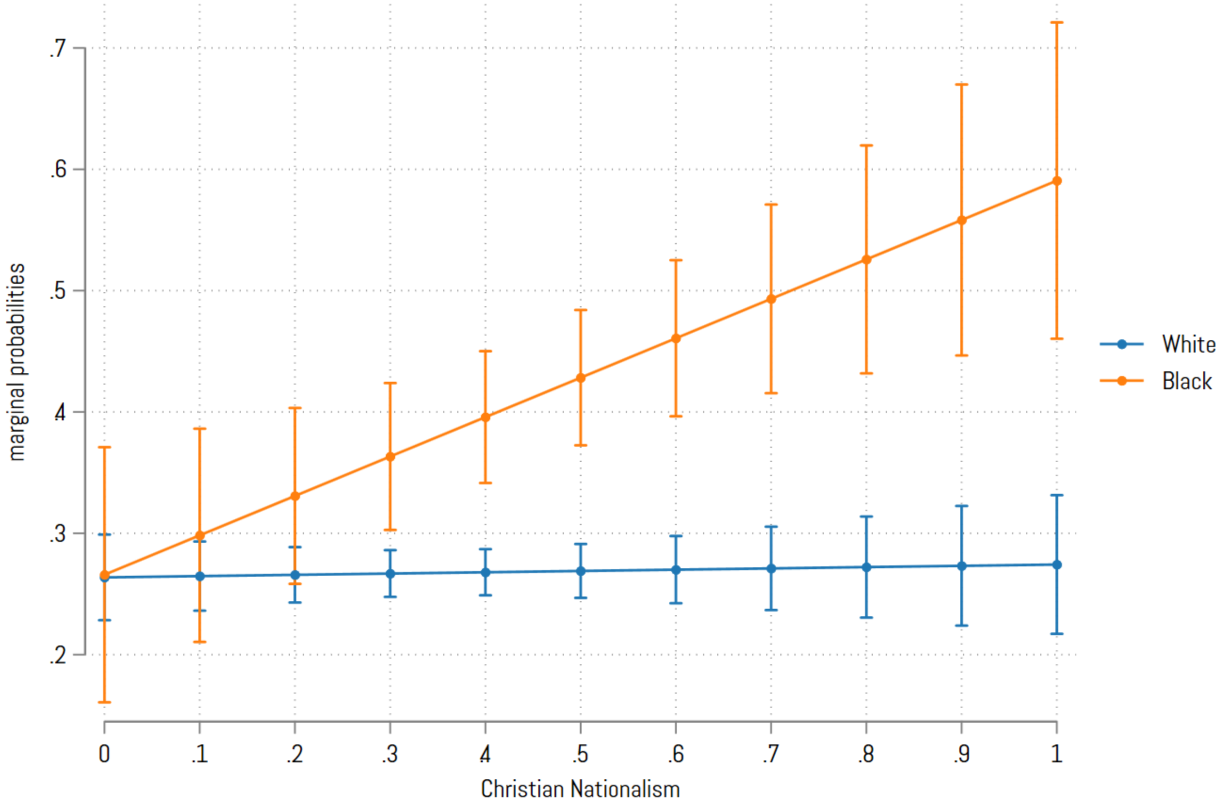

Figure S3: Predicted marginal probabilities of identifying with "progressive" for White and Black or Hispanic Americans across values of Christian nationalism. Error bars are 95% confidence intervals. Source: National Addiction and Social Attitudes Survey, Waves 1 and 3.

Panel A. Black Americans vs. White Americans

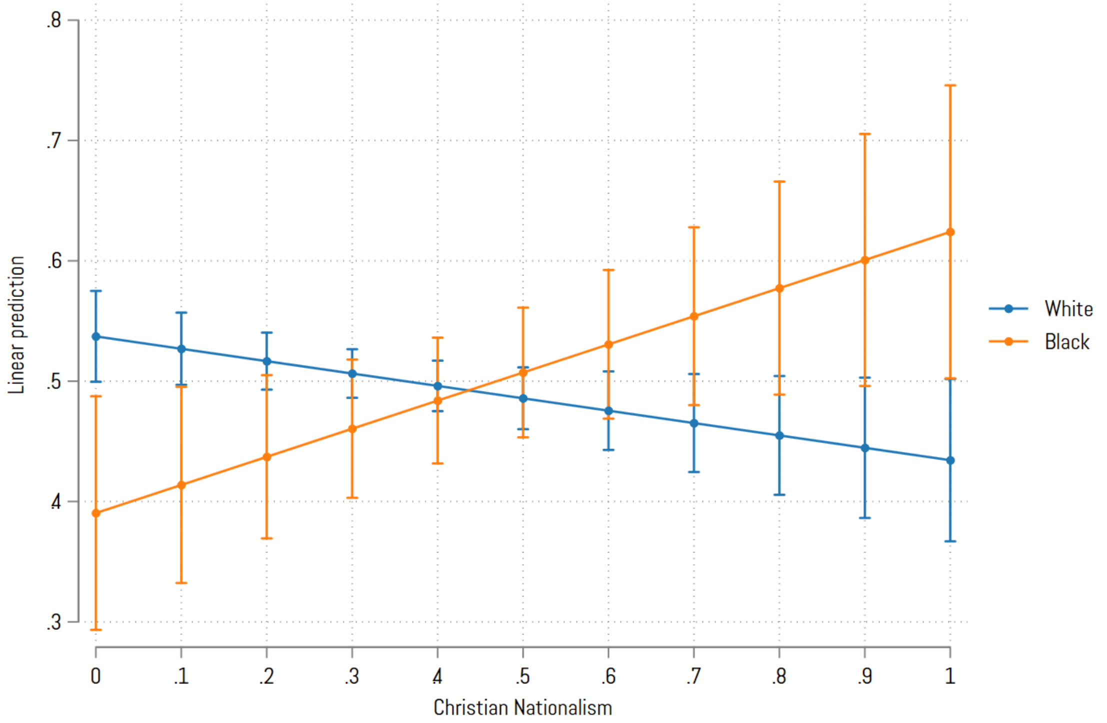

Panel B. Hispanic Americans vs. White Americans

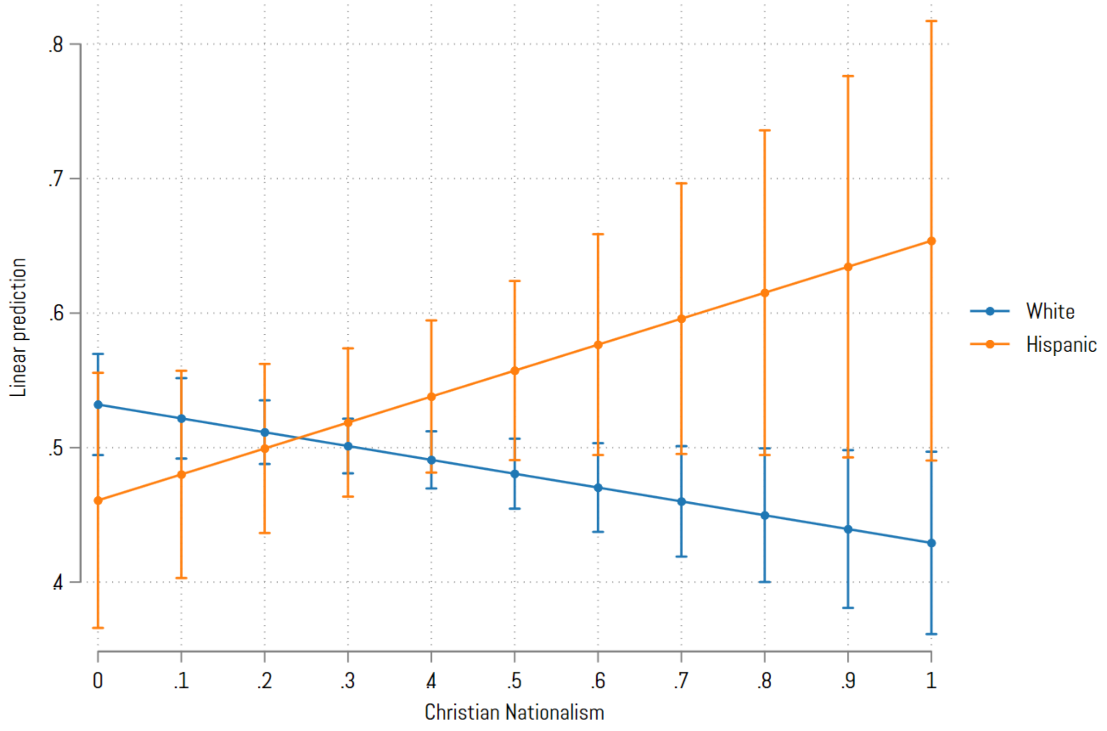

Figure S4: Marginal Contrasts of Christian nationalism and being Black vs. White on identifying as "woke" among Democrats, Republicans, and Independents. Error bars are 95% confidence intervals. The dotted red line represents the effect for White Americans for contrast. Source: National Addiction and Social Attitudes Survey, Waves 1 and 3.

Panel A. Democrats (Black vs. White)

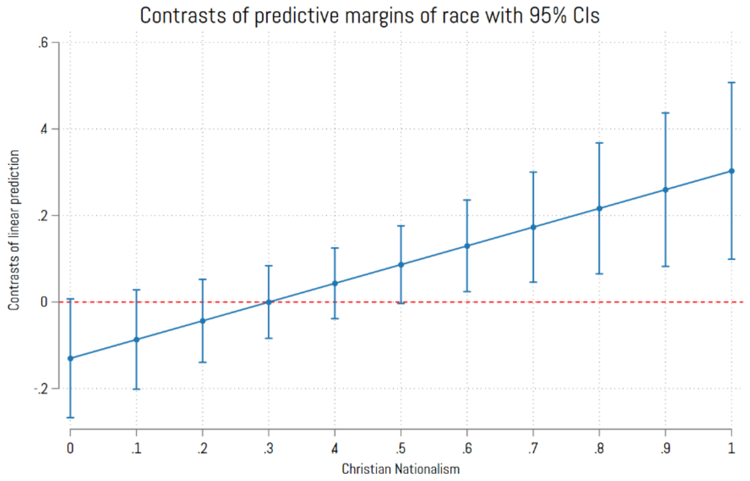

Pane B. Republicans (Black vs. White)

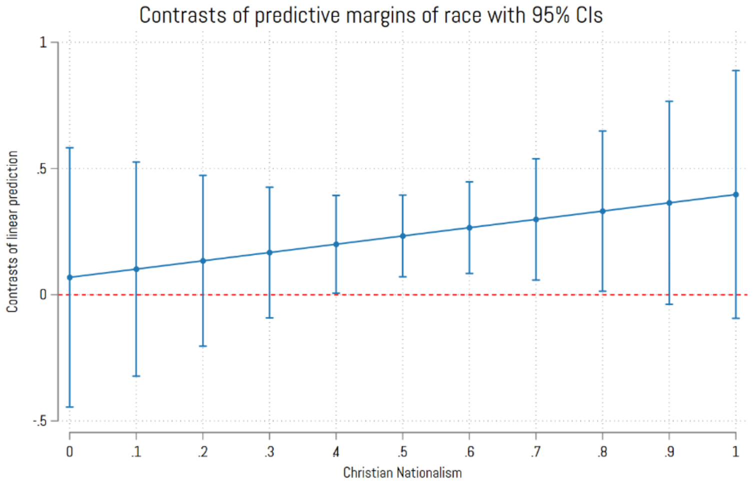

Panel C. Independents (Black vs. White)

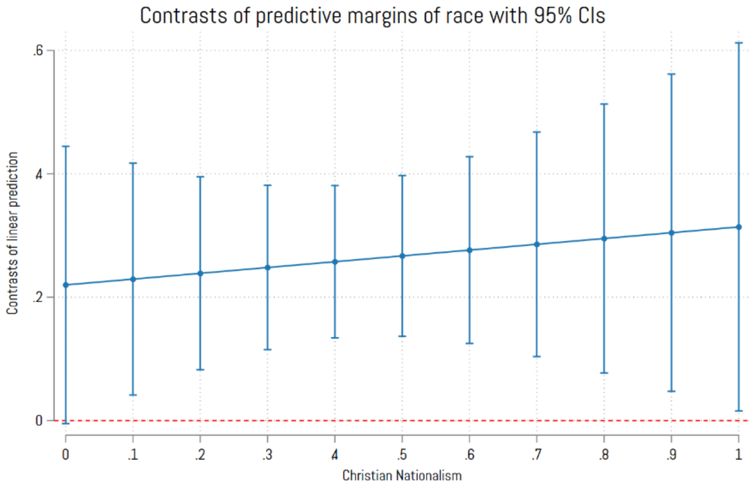

Figure S5: Marginal Contrasts of Christian nationalism and being Black vs. White on identifying as "progressive" among Democrats, Republicans, and Independents. Error bars are 95% confidence intervals. The dotted red line represents the effect for White Americans for contrast. Source: National Addiction and Social Attitudes Survey, Waves 1 and 3.

Panel A. Democrats (Black vs. White)

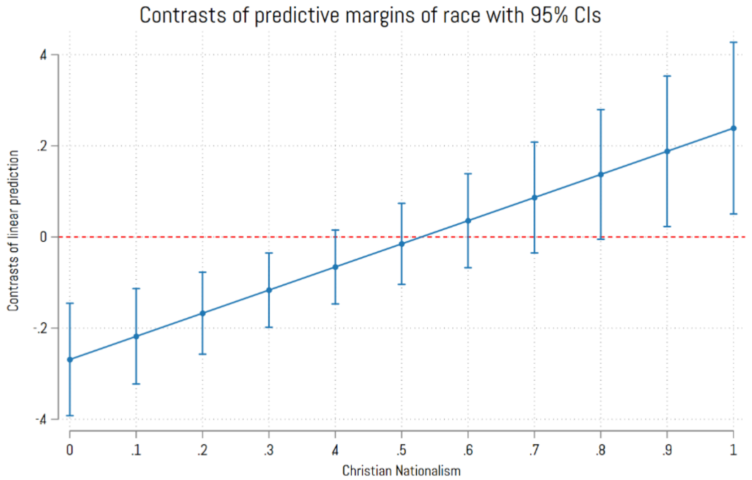

Panel B. Republicans (Black vs. White)

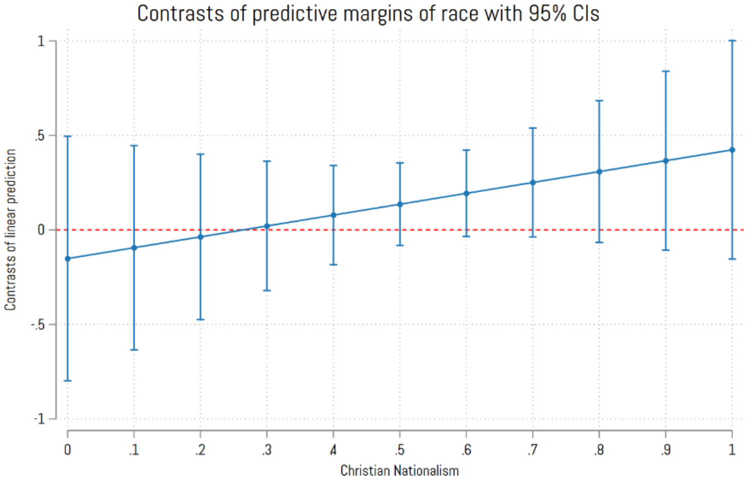

Panel C. Independents (Black vs. White)

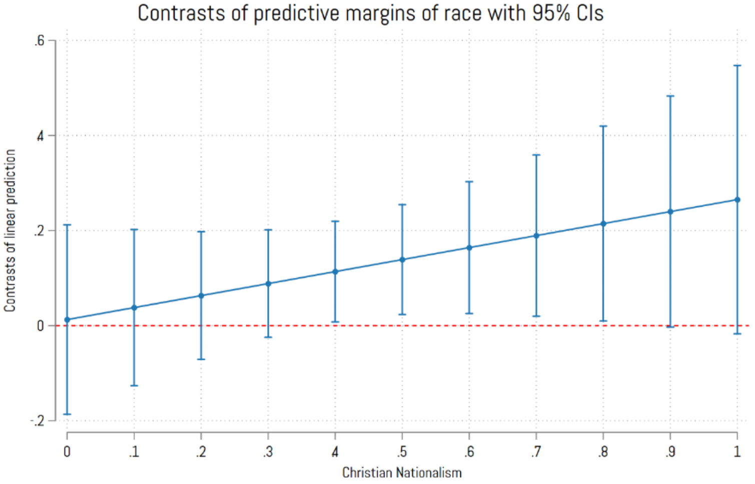

Figure S6: Marginal Contrasts of Christian nationalism and being Hispanic vs. White on identifying as "progressive" among Democrats, Republicans, and Independents. Error bars are 95% confidence intervals. The dotted red line represents the effect for White Americans for contrast. Source: National Addiction and Social Attitudes Survey, Waves 1 and 3.

Panel A. Democrats (Hispanic vs. White)

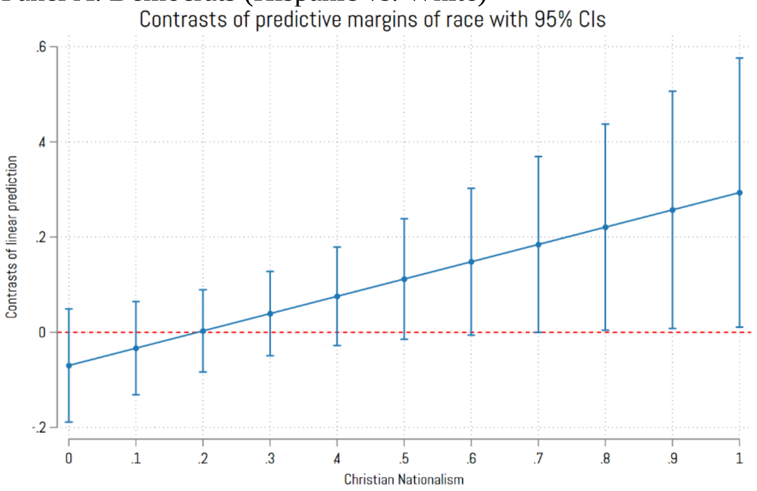

Panel B. Republicans (Hispanic vs. White)

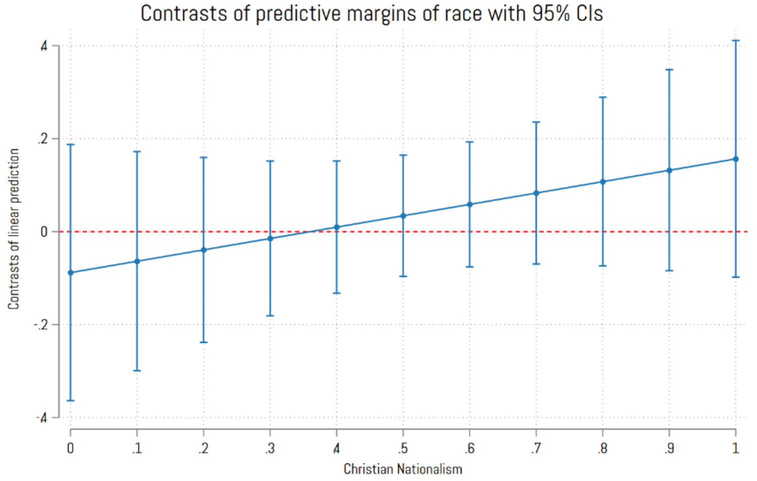

Panel C. Independents (Hispanic vs. White)

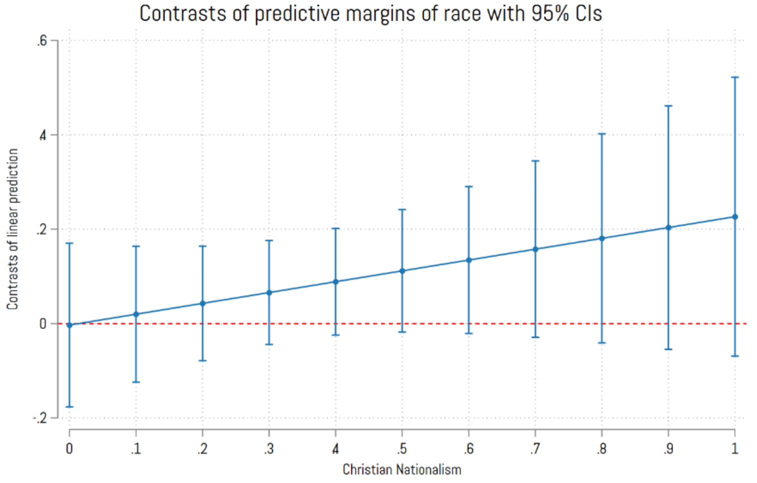

Supplement: nfaf009_Supplementary_Data [file nfaf009_supplementary_data.pdf]
